# Benchmarking the transparency, comprehensiveness and specificity of population nutrition commitments of major food companies in Malaysia

**DOI:** 10.1186/s12992-020-00560-9

**Published:** 2020-04-17

**Authors:** SeeHoe Ng, Gary Sacks, Bridget Kelly, Heather Yeatman, Ella Robinson, Boyd Swinburn, Stefanie Vandevijvere, Karuthan Chinna, Mohd Noor Ismail, Tilakavati Karupaiah

**Affiliations:** 1 Early Start, School of Health and Society, University of Wollongong, Northfields Avenue, Wollongong, NSW 2522 Australia; 2 Global Obesity Centre (GLOBE), Deakin University, 221 Burwood Highway, Burwood, VIC 3125 Australia; 3 School of Population Health, University of Auckland, 2 Morrin Road, Auckland, 1072 New Zealand; 4 School of Medicine, Faculty of Health and Medical Sciences, Taylor’s University, 47500 Subang Jaya, Selangor Malaysia; 5 Faculty of Social Sciences and Leisure Management, Taylor’s University, 47500 Subang Jaya, Selangor Malaysia; 6 Dietetics Programme, School of Healthcare Sciences, Faculty of Health Sciences, Universiti Kebangsaan Malaysia, 50300 Kuala Lumpur, Malaysia; 7 School of Biosciences, Faculty of Health and Medical Sciences, Taylor’s University, 47500 Subang Jaya, Selangor Malaysia

**Keywords:** Population nutrition, Obesity, Non-communicable diseases, Commitments, Food company, Accountability, Policy

## Abstract

**Background:**

The aim of this study was to assess the commitments of food companies in Malaysia to improving population nutrition using the Business Impact Assessment on population nutrition and obesity (BIA-Obesity) tool and process, and proposing recommendations for industry action in line with government priorities and international norms.

**Methods:**

BIA-Obesity good practice indicators for food industry commitments across a range of domains (*n* = 6) were adapted to the Malaysian context. Euromonitor market share data was used to identify major food and non-alcoholic beverage manufacturers (*n* = 22), quick service restaurants (5), and retailers (6) for inclusion in the assessment. Evidence of commitments, including from national and international entities, were compiled from publicly available information for each company published between 2014 and 2017. Companies were invited to review their gathered evidence and provide further information wherever available. A qualified Expert Panel (≥5 members for each domain) assessed commitments and disclosures collected against the BIA-Obesity scoring criteria. Weighted scores across domains were added and the derived percentage was used to rank companies. A Review Panel, comprising of the Expert Panel and additional government officials (*n* = 13), then formulated recommendations.

**Results:**

Of the 33 selected companies, 6 participating companies agreed to provide more information. The median overall BIA-Obesity score was 11% across food industry sectors with only 8/33 companies achieving a score of > 25%. Participating (*p* < 0.001) and global (*p* = 0.036) companies achieved significantly higher scores than non-participating, and national or regional companies, respectively. Corporate strategy related to population nutrition (median score of 28%) was the highest scoring domain, while product formulation, accessibility, and promotion domains scored the lowest (median scores < 10%). Recommendations included the establishment of clear targets for product formulation, and strong commitments to reduce the exposure of children to promotion of unhealthy foods.

**Conclusions:**

This is the first BIA-Obesity study to benchmark the population nutrition commitments of major food companies in Asia. Commitments of companies were generally vague and non-specific. In the absence of strong government regulation, an accountability framework, such as provided by the BIA-Obesity, is essential to monitor and benchmark company action to improve population nutrition.

## Background

Malaysia is among the countries with high obesity [1] and non-communicable disease (NCD) [2] rates in the South-East Asian region. The magnitude of risk for premature death from NCDs was 17% in Malaysia in 2016 [3]. ‘Dietary risks’ for NCDs in Malaysia account for 14.6% and ‘high body mass index’ accounts for 9.9% of disability-adjusted life years, as estimated by the *Global Burden of Disease* [4]. Key causes of unhealthy diets are rapid urbanisation, economic growth and social change coupled with trade liberalisation, which collectively trigger food system shifts towards convenience and ultra-processed foods [5–8]. Almost 70% of Malaysia’s population is urbanised [9] with increased market concentration of ultra-processed foods such as sweet and savoury snacks, carbonated drinks, packaged foods, biscuits and confectionery [6]. A recent population study in urban Malaysia highlighted that increased atherogenic and insulinemic risk profiles and obesity were associated with dietary patterns high in calories, fat, and sugars [10].

Prevention of diet-related NCDs requires consideration of the production, marketing, and consumption of commercially produced ultra-processed food products [11, 12]. The scope for preventive action for improving population nutrition therefore extends to actions by commercial food producers. The WHO [13, 14] recognises the need for transnational, regional and local food and non-alcoholic beverage industries, retailers, and catering companies to take responsibility in tackling obesity and diet-related NCDs via product reformulation, nutrition labelling, responsible marketing to children and healthy food accessibility. The Malaysian government, through its Eleventh Malaysia Plan 2016–2020, identified the private sector as a key stakeholder in promoting health, specifically through corporate social responsibility (CSR) activities [15].

Malaysia’s *National Plan of Action for Nutrition of Malaysia (NPANM) III 2016–2025* [16], and the *National Strategic Plan for Non-communicable Disease* (NSP-NCD) *2010–2014* [17] and *NSP-NCD 2016–2025* [18] set the basis for food industry engagement as part of efforts to improve population nutrition and health. In response to the NSP-NCD, the *Federation of Malaysian Manufacturers* (FMM) developed a range of related commitments and also engaged more companies to participate in NCDs prevention and control programmes [19]. Industry commitments included self-regulation approaches such as the *Responsible Advertising to Children* (Malaysia Pledge), and participating in the *Malaysian Healthier Choice Logo* programme and sugar reformulation initiative [19–21]. In addition, the Malaysian government has stated an intention to implement mandatory regulations such as imposing declarations for total sugars and sodium for all food products, restricting television advertising of foods and beverages high in fat, sugar and salt targeting children, and imposing a sugar tax on unhealthy foods and beverages [16]. Therefore, an independent monitoring framework is needed to generate baseline data to enable future comparisons if there is progress in implementing mandatory regulations.

Monitoring private-sector commitments to population nutrition and health [22] is critical to holding the food industry accountable for their role in efforts to improve population health. This initiative would foster evaluating the extent to which the ‘profit-only’ model of the food industry is shifting towards a ‘health viable profit’ model [23], whilst managing conflicts of interest in public-private partnerships [14, 24]. The Access to Nutrition Index (ATNI) [25] evaluates food and non-alcoholic beverage manufacturers’ commitments at a global level to reducing malnutrition and improving infant nutrition [25, 26]. A similar tool, the Business Impact Assessment - Obesity (BIA-Obesity) [27] uses less resource intensive methods to assess companies’ commitments in population nutrition and obesity at the national level. The BIA-Obesity assessment also includes quick service restaurants (QSR) and retailers such as supermarkets and convenience stores, in addition to food and non-alcoholic beverage manufacturers. The BIA-Obesity tool has previously been applied to Australia [28–30], New Zealand [31], and Canada [32]. It has been recommended that BIA-Obesity country level evaluations be used to monitor and evaluate food industry’s progress towards meeting country specific nutrition policies and health criteria, while at the same time building a central database to enable cross-country comparisons [27, 33].

We conducted the first BIA-Obesity in an Asian country. The study included those food companies with national, regional and global presence and with the largest market shares in Malaysia, for each of the four sectors (food and non-alcoholic beverage manufacturing, QSR and retailer sectors). The study also generated recommendations for industry actions, in line with government’s priorities and international norms.

## Methods

### Business impact assessment on population nutrition and obesity (BIA-obesity)

BIA-Obesity is a defined tool and process developed by the International Network for Food and Obesity/ NCDs Research, Monitoring and Action Support (INFORMAS) [22, 27]. It utilises a step-based approach to assess the nutrition-related practices of major food companies within a country’s food system. Phase I of BIA-Obesity focuses on an assessment of company policies and commitments, while Phase II focuses on company practices, including how commitments translate into actions. This study implemented and reported Phase I of BIA-Obesity in Malaysia.

### Adaptation of the tool

The development of BIA-Obesity has been detailed by Sacks et al. [27]. In brief, the BIA-Obesity assessment incorporates six domains: (1) *Corporate strategy* - assesses company’s overarching approach to addressing obesity and NCDs; (2) *Product formulation* – assesses targets of nutrients of concern, and portion size or energy reduction in new or existing products; (3) *Nutrition labelling* – focuses on the display of nutrition information on packaged foods, online and/or menus, where applicable; (4) *Promotion practices* – benchmarks efforts to reduce marketing of non-core foods that do not fulfil specific nutrition criteria in all settings (including catalogues and in-store promotion in retailer sector), (5) *Product accessibility* – analyses availability and pricing commitments on healthy products, compared to non-core products; and (6) *Relationships with external organisations* – evaluates funded and supported corporate social responsibility (CSR) activities. Weighting of the domains used for this assessment was based on several consultations within the INFORMAS network [27]. The allocations are out of 100 as per sectors (see Additional file 1). Scoring was based on the comprehensiveness, transparency and specificity of policies and commitments.

**Fig. 1:**
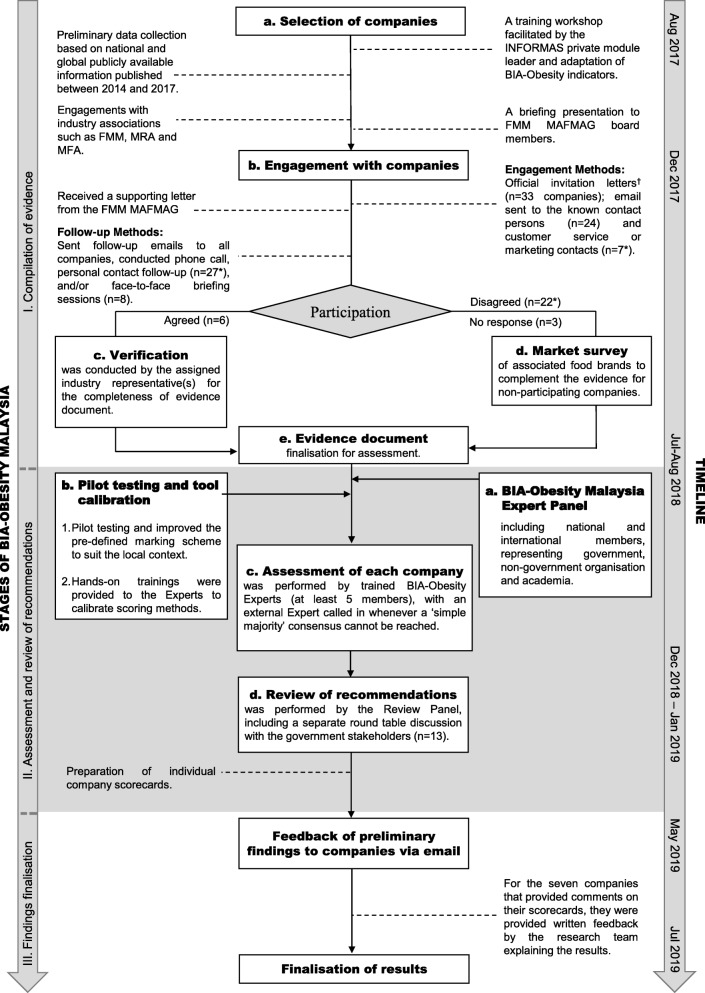
Process of implementation of BIA-Obesity in Malaysia. Abbreviations: BIA-Obesity = Business Impact Assessment-Obesity; FMM = Federation of Malaysian Manufacturers; FMM MAFMAG = Federation of Malaysian Manufacturers Malaysian Food Manufacturing Group; MRA = Malaysia Retailers Association; MFA = Malaysian Franchise Association. Notes: ^†^Invitation letters were posted to all companies except for 3 companies who did not have a maling address. For these companies, communication was via email. *Three companies shared the same parent company and for these the communication was directed to the parent company

The Malaysian research team adapted the global version of the BIA-Obesity tool for the local context, in conjunction with the INFORMAS private sector module leader (GS). The process of adapting the tool consisted of an iterative process that included multiple face-to-face training workshops on the BIA-Obesity protocol and discussion amongst the project team. The process for implementation of BIA-Obesity in Malaysia consisted of three stages: compilation of evidence, assessment and review of recommendations, and findings finalisation (Fig. 1).

The BIA-Obesity tool was adapted with several modifications on selected indicators to suit the local context [27]. For instance, BIA-Obesity Malaysia included modifications to assess: (1) voluntary adoption of Malaysian *Healthier Choice Logo* (product formulation and labelling domains), (2) implementation of quantitative ingredient declarations (QUIDs) (labelling domain); (3) government-endorsed front-of-pack (FOP) labelling scheme (i.e. a single icon for ‘energy based on a daily calorie intake of 2000 kcal and *Healthier Choice Logo*) (labelling domain); (4) specifying policies related to (i) nutrient function claims and (ii) nutrition claim in accordance with permitted claims in Malaysia Food Regulations 1985 (labelling domain) (see Additional file 2).

### Stage I: Compilation of Evidence

- *Selection of companies*

As per the BIA-Obesity protocol [27], the most prominent food companies for each sector (food and non-alcoholic beverages, QSR, and retailers) in Malaysia were selected for assessment based on market share information from the Euromonitor Passport database for 2016 [34–38]. Market share information was based on the retail value (measured based on the Passport database’s retail selling price, RSP) for major market sectors but excluding data for unrelated sub-categories (e.g. minimally processed foods such as cooking oils, rice, and mineral waters; and specialty foods like infant formula). The rationale for this exclusion is in tandem with the BIA-Obesity protocol [27], which aims to identify prominent food companies with the greatest influence on the food environment in Malaysia, giving opportunity to improve population diets. In addition, the tool focused on initiatives with regards to obesity prevention, which excludes undernutrition issues (e.g. fortified products).

Market share according to food categories (except retailer sector) was applied for company selection (see Additional file 3). The retail values of companies per food category were first ranked from the highest to lowest. Subsequently, company selection was based on two criteria: (1) at least the top quarter of retail values for each subcategory; and (2) sum of the average retail value for all subcategories to fulfil at least 50% of the relevant market share. The second criterion however was not applied to the retailer sector in Malaysia due to the large number of smaller grocers and the relatively small market share of the largest companies in the sector. Thirty-three food companies were selected, including food and beverage manufacturers (*n* = 22, representing 62.9% of the relevant market share), QSR (*n* = 5, 79.1% market share) and retailer (*n* = 6, 26.2% market share) sectors (Table 1). Most companies selected for inclusion in the study had their parent company located outside of Malaysia (25/33), although six companies were publicly listed in the Malaysian stock market [39].

**Table 1:** Characteristics of the selected companies (*n* = 33) across sectors

No.	National company name	Assigned name	Market share* (%)	Characteristics of the company	Category/ sub-category*
**Manufacturer sector (total market share = 62.9%)**					
1.	Fraser & Neave Holdings Bhd.	Fraser & Neave	11.3	Regional company^†^	Ice-cream, drinking milk products, other dairy, carbonates, concentrates, juice, RTD Tea, sports and energy drinks, and Asian specialty drinks
2.	Nestlé (M) Bhd.	Nestlé	10.9	Global company^†^	Confectionery, ice-cream, RTE cereal, instant noodles, drinking milk products, yoghurt products, other dairy, and RTD coffee
3.	Yeo Hiap Seng (M) Bhd.	Yeo Hiap Seng	6.0	Regional company	Ready meals, processed meat and seafood, spread, instant noodles, drinking milk products, juice, and RTD tea, and Asian specialty drinks
4.	Mondelēz (M) Sales Sdn. Bhd.	Mondelēz	4.0	Global company	Biscuits, confectionery, savoury drinks, and cheese
5.	Etika Group of Companies	Etika Group	3.7	Global company	Other dairy, carbonates, juice, RTD coffee, RTD tea, and sports and energy drinks
6.	Campbell Soup SEA Sdn. Bhd.	Campbell’s Soup	3.4	Global company	Biscuits, and soup
7.	Malaysia Milk Sdn. Bhd.	Malaysia Milk	3.3	Regional company	Drinking milk products, yoghurt products, other dairy, juice, and RTD tea
8.	Unilever (M) Holdings Sdn. Bhd.	Unilever	3.2	Global company	Ice-cream, ready meals, soup, and spread
9.	Coca-Cola Malaysia	Coca-Cola	3.1	Global company	Carbonates, juice, and RTD tea
10.	Fonterra Brands (M) Sdn. Bhd.	Fonterra	2.2	Global company	Cheese, drinking milk products, yoghurt products, and other dairy
11.	Kellogg Asia Marketing Inc.	Kellogg’s	2.2	Global company	Savoury snacks and RTE cereal
12.	Barkath Co-Ro Mfg Sdn. Bhd.	Barkath Co-Ro	1.6	Global company	Concentrates
13.	Dutch Lady Milk Industries Bhd.	Dutch Lady	1.5	Global company^†^	Drinking milk products and yoghurt products
14.	Mamee-Double Decker (M) Sdn. Bhd.	Mamee	1.4	National company	Savoury snacks, instant noodles, and yoghurt products
15.	Gardenia Bakery KL Sdn. Bhd.	Gardenia	1.2	Regional company	Baked goods and spread
16.	Hup Seng Perusahaan Makanan (M) Sdn. Bhd.	Hup Seng	0.8	National company^†^	Biscuits and savoury snacks
17.	Munchy Food Industries Sdn. Bhd.	Munchy’s	0.8	National company	Biscuits and savoury snacks
18.	Ferrero SpA	Ferrero	0.6	Global company	Confectionery and spread
19.	Clouet & Co (KL) Sdn. Bhd.	Ayam Brand	0.6	Global company	Processed meat and seafood, and juice
20.	The Italian Baker Sdn. Bhd.	Massimo	0.6	National company	Baked goods
21.	Ayamas Food Corp Sdn. Bhd.	Ayamas	0.3	National company	Processed meat and seafood
22.	Ramly Food Processing Sdn. Bhd.	Ramly	0.3	National company	Processed meat and seafood
**Quick service restaurant sector (total market share = 79.1%)**					
23.	QSR Stores Sdn. Bhd. (Pizza Hut)	Pizza Hut	26.3	Global company	Pizza consumer foodservice
24.	QSR Stores Sdn. Bhd. (KFC)	KFC	21.0	Global company	Fast food
25.	Dommal Food Services Sdn. Bhd. (Domino’s)	Domino’s	15.8	Global company	Pizza consumer foodservice
26.	Gerbang Alaf Restaurants Sdn. Bhd. (McDonald’s)	McDonald’s	13.8	Global company	Fast food
27.	Golden Donuts Sdn. Bhd. (Dunkin’ Donuts)	Dunkin’ Donuts	2.4	Global company	Fast food
**Retailer sector (total market share = 26.2%)**					
28.	GCH Retail (M) Sdn. Bhd.	Giant	9.3	Global company	Food retailer
29.	Tesco Stores (M) Sdn. Bhd.	Tesco	7.0	Global company	Food retailer
30.	7-Eleven Malaysia Sdn. Bhd.	7-Eleven	3.4	Global company^†^	Convenience store chain
31.	AEON Group	Aeon Group	2.9	Global company^†^	Food retailer
32.	Econsave Cash & Carry Sdn. Bhd.	Econsave	2.6	National company	Food retailer
33.	Mydin Mohamed Holdings Bhd.	Mydin	1.0	National company	Food retailer

*Abbreviations*: *RTD* ready-to-drink, *RTE* ready-to-eat

*Market share was extracted from Euromonitor Passport datasets Year 2016 [34–38] as per the retail selling price values of investigated category/ sub-category

^†^At least one subsidiary publicly listed company in the Malaysian stock market [39]

*Notes*

1. Definitions of the characteristics of the company

a. A global company is defined as providing goods or service worldwide across regions with its headquarters or parent company located outside Malaysia

b. A regional company may be a food and/or beverage business operating within the South-East Asian (SEA) region with its headquarters or parent company located outside Malaysia but within any of the SEA countries

c. A national company is denoted as a company mainly distributing its goods or services within Malaysia and its headquarters or parent company located in Malaysia

2. Etika Group of Companies included Etika Beverages Sdn. Bhd. (manufacturer for soft drinks) and Etika Dairies Sdn. Bhd. (manufacturer of other dairy namely condensed or evaporated milk)

3. AEON Group included AEON Big (M) Sdn. Bhd. and AEON Co. (M) Bhd. (a listed company in the Malaysian stock market)

4. QSR Brands (M) Holdings Bhd. included QSR Stores Sdn. Bhd. (Pizza Hut and KFC) and Ayamas Food Corp Sdn. Bhd

Company commitments against assessed indicators published between 2014 and 2017 were extracted from publicly available information. Evidence was sourced from company/ brand websites, annual reports, policy statements or guidelines, press releases and social media posts (e.g. Facebook). The study included information published at the national, global industry association and/or parent company level, and government websites. Each company’s commitments were then compiled in a Microsoft Word file.

b. *Engagement with companies*

Various industry associations were contacted but only FMM was willing to support the project and circulated an endorsement letter to members. Contact information for individual companies was collated from FMM (*n* = 22) and also by accessing company/brand websites, phone call inquiries and/or professional networking websites, such as LinkedIn. The engagement process included sending official invitation letters to companies (*n* = 33); and emails to known contact persons (*n* = 24) and customer service or marketing contacts (*n* = 7) (see Additional file 4). Information related to the research purpose and process, industry’s role, risks and benefits, as well as the use of research outcomes were provided to the selected companies. Subsequent follow-up emails, phone calls and/or individual briefing sessions (*n* = 8) were conducted in an effort to increase company participation by providing more information about the study.

c. *Verification*

Company-specific evidence in a summarised document was sent to each of the participating company contacts. Participating companies were those that assigned a representative(s) to verify the evidence document, as well as provide additional evidence if available and substantiated. In total, six participating companies went through this process. The verification process took 3 to 5 months. Participating companies returned a verification sign-off form and there was a non-disclosure clause to keep confidential statements disclosed solely for scoring purposes.

d. *Market survey*

Non-participating companies were those which did not consent to verify the evidence document. Their assessment was consequently based on publicly available information. This is consistent with other studies [26, 28–30, 32, 40]. Market surveys of non-participating companies were conducted on a sample of in-store products of food and beverage manufacturers, menus for QSR and in-house brands for retailer sectors. Photographs of products of these companies were captured to provide supplementary evidence for selected indicators of the nutrition labelling domain. These included availabilities of QUIDs; total sugars, added sugars or *trans*-fat content on back-of-pack labelling; types of FOP labelling; and/or menu labelling.

e. *Evidence document*

With the completion of the data collection process for participating and non-participating companies, the finalised evidence documents were formatted as per indicators for each company to be evaluated. Evidence was consolidated for national and global commitments. These finalised evidence documents underwent the review process as outlined in Stage II.

### Stage II: Assessment and Review of Recommendations

- *BIA-Obesity Malaysia Expert Panel*

A panel of experts (Expert Panel) was established to perform the assessment of company commitments as per the BIA-Obesity scoring protocol. Selection criteria for experts included area of expertise (e.g. public health, nutrition policy), absence of any self-declared conflict of interest (i.e. no formal collaborations with food and beverage companies) and no involvement in similar studies. The invited Expert Panel represented government, non-government organisations (NGO) and academia (local and international).

Eight of ten invited experts consented to join the Expert Panel and one expert with a declared conflict of interest was subsequently rejected. The composition of the Expert Panel (*n* = 7) is described in Table 2A. The members had more than 10 years’ experience in their field and were from academia (*n* = 4), government (*n* = 2) and an NGO (*n* = 1). Their combined expertise covered public health nutrition (*n* = 3), national nutrition policy development (n = 4), and public affairs management (n = 1). Prior to the assessment, each member signed a non-disclosure form and agreed to fulfil all confidentiality obligations.

b. *Pilot testing and tool calibration*

**Table 2:** Sociodemographic data of panels as part of BIA-Obesity Malaysia process of assessment

Characteristics	A	B
	Expert Panel^b^ (n)	Review Panel^c^ (n)
Age (years)		
20–39	1	3
40–59	3	10
60 or above	3	–
Gender		
Male	2	2
Female	5	11
Education level		
Degree	2	7
Master	1	6
PhD	4	–
Professional Background		
Academia/ professionals	4	–
Non-government/ non-profit organisation	1	–
Government stakeholder	2	13
Working Experience (years)		
5–10	–	3
11–20	4	7
21–30	1	3
31 or above	2	–
Expertise^a^		
Public affairs management (e.g. corporate, inter-agency collaboration including private sector)	1	2
Public health nutrition (e.g. nutrition promotion, food marketing, food labelling, food education programme evaluation)	3	7
National policy development (e.g. national nutrition plan, national food and nutrition policy, food regulations, obesity and/or NCDs prevention)	4	5
Food regulations and/or food safety auditing	–	3

*Abbreviations*: *NCDs* non-communicable diseases, *PhD* Doctor of Philosophy

*Notes*:

^a^More than one field of expertise may be stated by the Expert or Review Panel.

^b^Seven members of the Expert Panel (including an academic who refereed whenever the 'simple majority rule' failed) performed the assessment.

^c^The Review Panel comprised the Expert Panel and thirteen government stakeholders who formulated the recommendations. This column only presents the profile of the thirteen government stakeholders involved in the round table session

As part of the iterative process of adapting the BIA-Obesity tool to the Malaysian context, pilot testing of the assessment criteria was conducted by two experts. Based on the pilot assessments of one company, the scoring scheme was revised before proceeding with a training session for all Expert Panel members to further calibrate the tool. In this process, the scoring scheme was further revised, for example, to include results of the market survey and to add the *Healthier Choice Logo* as a criterion for assessment of product healthiness.

c. *Assessment of each company*

Within a 4-month period, a minimum of five members of the Expert Panel completed scoring of all companies using the BIA-Obesity Malaysia tool. Communications via emails and video conferencing maximised discussion between the researchers and Expert Panel. Each member scored the evidence independently. Upon consolidating the Expert Panel scores, outliers (i.e. weighted overall score > 1.5 times of the interquartile range) were returned to relevant expert(s) for re-consideration.

The final score for each indicator was determined based on a ‘simple majority rule’ such as at least 4 out of 6 members in the Expert Panel casting the same score. In the event that a ‘simple majority rule’ could not be reached (e.g. where the same number of experts allocated scores to two different values), an additional academic referee was called in to make the final decision. Baharad et al. [41] indicated that ‘simple majority rule’ was common to organisations in making decisions and adding another voter would be preferred to removing an existing competent voter to reach consensus. Table 3 shows examples of publicly available commitments using illustrative quotes, scoring criteria, and their corresponding scorings, by domain.

d. *Review of recommendations*

**Table 3:** Examples of publicly available commitments and their scorings for BIA-Obesity Malaysia

Domain	Indicator	Example commitment	Scoring criteria	Score
Corporate Strategy	Does the company have an overarching commitment to population nutrition and health articulated in strategic documents (e.g., mission statement, strategies, or overarching policies)?	“… we have been in the country for more than… Our key commitments: (a) Launch more nutritious foods and beverages, especially for mothers-to-be, new mothers and children; (b) further decrease sugars, sodium and saturated fat in our foods and beverages; (c) apply and explain nutrition information on packs, at point of sale and online; (d) offer guidance on portions for our products; (e) market to children only choices that help them achieve a nutritious diet…”	10: Yes, a national-level commitment, publicly available 7.5: Yes, a global-level commitment, publicly available 5: Yes, a national- or global-level commitment, but not publicly-available OR some commitment but weak in nature 0: No clear commitments to improving population nutrition and health	10
Product formulation	Does the company publish a comprehensive set of commitments or objectives related to new product development and reformulating its existing products with respect to reducing the nutrients of concern and energy (salt, saturated fats, trans fats, added sugar and kilojoules)?	“It also has on-going efforts to provide healthier options with reduced sugar, fat and sodium content via its ‘Lite’ range, a growing organic selection, variants that suit a plethora of tastes from mild to super spicy, whilst innovating in terms of packaging.”	10: Yes, specific national-level commitments/ objectives that are publicly available or specific global commitments/objectives that include specific reference to the country or market in question 7.5: Yes, specific global commitments/ objectives that could specifically apply to the country in question that are publicly available 5: Has specific national-level commitments/ objectives, but not publicly available (e.g. disclose to INFORMAS team). 2.5: Has national or global-level commitments/ objectives in this area that are available publicly, but these commitments/ objectives are vague and non-specific 0: No	2.5
Nutrition labelling	Does the company commit to use a ‘comprehensive’* FOP labelling system? *‘Comprehensive’ refers to beyond a single icon for ‘energy’ e.g. traffic lights, warning labels, healthy stars, healthier choice logo and etc.	“The Healthier Choice Logo (HCL) was launched in Malaysia by the Ministry of Health. It is in line with the National Plan of Action for Nutrition Malaysia (NPANM) III (2016–2025) to promote healthy eating and active living for all. This initiative is based on the same principles as the Choice labelling programme and other voluntary labelling programmes around the world… (the company) is committed to delivering the tastiest and healthiest product options in every category… To-date, (the company) has 41 recipes that are certified with HCL logo.”	10: Symbols or logos (e.g. health stars, traffic light, warning labels, etc.) that indicate healthy products, applied across all product categories 7.5: Symbols or logos (e.g. national endorsed system - Healthier Choice Logo, Healthier Choice Symbol) that indicate healthy products, applied across some product categories. 5: Numeric information on levels of key nutrients (e.g. sodium, total fat, saturated fat or total sugar) applied across all product categories, 2.5: Numeric information on levels of key nutrients (e.g. sodium, total fat, saturated fat or total sugar) applied across SOME product categories, 0: No FOP labelling OR energy FOP labelling.	7.5
Promotion practices	To what age group(s) does the broadcast marketing policy apply?	“… we will not address advertising communications to audiences consisting primarily of pre-school age children, i.e. those who are younger than six years old.”	10: 18 years and/ or under 8: 16 years and/ or under 6: 14 years and/ or under 4: 12 years and/ or under 2: Under 10 years 0: No policy / no information	2
Product accessibility	Does the company’s policy position support WHO’s position on fiscal policies to make healthier foods relatively cheaper and non-core foods relatively more expensive, as articulated in the WHO Global Action Plan for NCDs and the Report of the Commission on Ending Childhood Obesity, Recommendation 1.2)?	“… changes in tax laws and unanticipated tax liabilities could adversely affect the taxes we pay and our profitability… Any increases in income tax rates, changes in income tax laws or unfavourable resolution of tax matters could have a material adverse impact on our financial results.”	10: Strong support (e.g., includes support for taxes on non-core foods, broadly defined, as well as subsidies for healthy foods) 5: Weak support (e.g., includes support for taxes on non-core foods, narrowly defined, or subsidies for healthy foods) 0: No details available −10: Strongly opposed (e.g., opposes soft drinks tax or non-core foods tax OR both taxes)	−10
Relationships with external organisations	Does the company publish details of its political donations? (when not prohibited by government policy)?	“According to the local Code of Business Ethics Conducts, “no political contribution (i.e. such as funds, assets and gifts) shall be made by or on behalf of the Company”.”	10: Yes, information on national-level activity is publicly available (on a company website or document) OR declaration of no activity in this area 5: Yes, global policy (i.e. does not specifically mention Malaysia) and disclose to INFORMAS team that it applies in Malaysia. 0: No	10

*Abbreviations*: *FOP* front-of-pack, *HCL* Healthier Choice Logo, *INFORMAS* International Network for Food and Obesity/ NCDs Research, Monitoring and Action Support, *NCDs* non-communicable diseases, *WHO* World Health Organization

The research team developed preliminary recommendations for each company based on Expert Panel assessment of their policies. A Review Panel was established to review and harmonise these recommendations to ensure that these were consistent with national policies, government directions and international standards, as well as achievable under the local conditions. The Review Panel included the Expert Panel members, with additional government stakeholders. Selection criteria for these government stakeholders included regular engagement with food industry on policy implementation matters related to food reformulation, labelling, promotion and/or accessibility, absence of any self-declared conflict of interest, and consenting to attend a discussion.

Hence, in addition to the Expert Panel members, 13 government stakeholders were included on the Review Panel tasked with reviewing the recommendations. Of note, government stakeholders all had a minimum 5 years of working experience with combined expertise of inter-agency collaboration with the private sector (*n* = 2), food regulation and/or food safety auditing (*n* = 3), nutrition policy planning specific to obesity and/or NCD prevention (*n* = 5), and/or public health nutrition (*n* = 7) (Table 2B).

### Stage III: Findings Finalisation

Preliminary scorecards were disseminated to the companies for verification, within a two-week period (see Additional file 5). Comments received from companies were addressed by minor amendments to stated key strengths and company-specific recommendations. All companies were invited to attend a closed-door meeting, held a month later, where overall findings of BIA-Obesity Malaysia were presented. Fifteen companies attended the meeting. The full technical report was publicly disseminated at a later date.

### Data analysis

Intraclass correlation coefficient (ICC) of the Expert Panel were determined using two-way random model and absolute agreement type across all the indicators as per company assessed. Agreement test of assessed indicators reaching ‘simple majority rule’ was calculated. *Kruskal Wallis* testing further examined the weighted score differences between the Expert Panel members, by domains and overall weighted scores. Differences in overall weighted scores were compared between participating and non-participating companies, and according to characteristics of companies (e.g. global vs regional and national companies, and listed *vs* non-listed companies in the Malaysian stock market [39]) using Mann-Whitney tests. For this study’s purpose, a global company is defined as one that provides goods or service worldwide across regions with its headquarters or parent company located outside Malaysia. Whereas, a regional company may be a food and/or beverage business operating within the South-East Asian (SEA) region with its headquarters or parent company located outside Malaysia but within any of the SEA countries. In contrast, a national company is denoted as a company mainly distributing its goods or services within Malaysia and its headquarters or parent company located in Malaysia. Finally, the association of market shares and overall weighted scores were tested using Spearman rank coefficient. IBM SPSS version 21.0 (SPSS Statistics Inc., Chicago IL, USA) was used to perform these analyses, with *p* < 0.05 set as the threshold for statistical significance.

## Results

Six out of 33 companies agreed to participate in the BIA-Obesity assessment process. Twenty-four companies declined to participate, while three did not respond. Some declined companies provided reasons for non-participation such as resource limitations (*n* = 2), lack of priority as perceived by senior management (*n* = 3), lack of local staff with relevant skills (e.g. nutritionist) to help with evidence collection (n = 2), tight schedules (n = 3), restriction in the company policy for public disclosure (*n* = 1), and scepticism regarding the assessment (n = 2).

### Consistency between Expert Panel Scorings

In terms of consensus between the Expert Panel members, the ICC for 33 companies ranged from 0.83 (95%CI 0.75, 0.89) to 0.99 (95%CI 0.98, 0.99). The agreement test of assessed indicators reaching ‘simple majority rule’ was 94.2% (i.e. 1787 out of 1897 applicable indicators assessed). There was no significant difference in weighted scores by domain between the Expert Panel for all assessed companies (*p* > 0.05).

### Overall weighted score

**Fig. 2:**
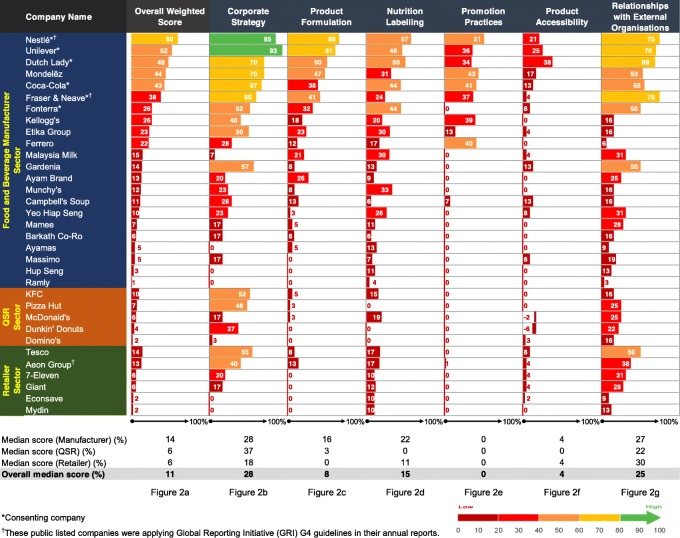
Overall weighted score (**a**) and percent of the total number of points by domain (**b**-**g**). Notes: 1. The negative scorings in **f** were deficit points after taking into consideration of the substantial evidence that the assessed company strongly opposed unhealthy food tax. This was assessed under the indicator – “Does the company’s policy position on fiscal policies to make healthier foods relatively more expensive, as articulated in the *WHO Global Action Plan for NCDs* and the *Report of the Commission on Ending Childhood Obesity*, Recommendations 1.2”. 2. The Expert Panel considered two non-participating companies (Etika Group and McDonald’s) showing no explicit evidence published between 2014 and 2017 for the implementation of the *Malaysia Pledge*. The cited comments were low transparency in reporting, no publicly available individual action plans and nutritional standards, and dynamic change in business ownership or licensees’ obligations

The overall weighted score for companies varied from 1% (Ramly) to 60% (Nestlé) (Fig. 2**a**). The overall median score was 11% across food industry sectors, but individual sector comparisons indicated that the median score for food and beverage manufacturers (14%) was greater than that of QSR and retailer sectors (both recorded as 6%).

Market share of companies was not significantly associated with the overall weighted scores (*r* = 0.20, *p* = 0.266). Neither was the overall weighted score difference between listed and non-listed companies in the Malaysian stock market (25.5% vs 10.3%; *U* = 57.0; *p* = 0.263). The median score of participating companies was significantly higher compared with non-participating companies (45.1% vs 6.9%; *U = 3.0; p* < 0.001). Global companies scored significantly higher, compared with regional and national companies (13.0% vs 5.8%; *U* = 70.0; *p* = 0.036).

The following sections describe domain-specific scorings expressed as percent of the total number of points, and the recommendations across sectors. As some indicators were not applicable for some industry companies (e.g. setting a trans-fat target was not applicable to beverage companies), the number of eligible companies varied per indicator.

The recommendations were developed based on collated constructive opinions of the Review Panel, which were generated from discussions on scorings for indicators and consideration of other research (e.g. BIA-Obesity Australia and New Zealand), WHO recommendations, and/or national nutrition plans. The Review Panel also considered differences between food companies global versus local commitments and disclosures (where relevant). Feasibility of recommendations in the Malaysian context, national norms and industry capability were also important considerations during the development of recommendations.

### Corporate strategy

The overall median score (28%) was highest for the corporate strategy domain with scores ranging between 0 and 93% (Fig. 2b). Twenty-eight out of 33 companies had policies or statements that included population nutrition and health as part of their business strategy. Companies with higher scores for this domain published their commitments aligning with international agendas (e.g. 2030 Agenda for SDGs, World Health Organizations (WHO) recommendations) and/or national government policies (e.g. NPANM III 2016–2025 or supported *Healthier Choice Logo* programme). However, most (22/33) scored < 50% and this lower score was attributed to weak commitments that were non-specific to the Malaysian context, coupled with irregular reporting.

Key recommendations were to (1) refer national and international recommendations (e.g. NPANM III 2016–2025, WHO Global NCD Action Plan, SDGs, etc.) when formulating targets and plans; (2) link targets to Key Performance Indicators of senior managers; and (3) report the progress at national level and on a regular basis. Whenever possible, the Review Panel encouraged companies to employ nutritionists, dietitians or equivalent professionals in their business. They also observed the low levels of engagement with the QSR sector with respect to implementation of nutrition policies in the past. Accordingly, the Review Panel called for greater engagement from food companies in regards to implementation of government-led initiatives for improving population nutrition and health.

### Product formulation

The overall median score for the product formulation domain was 8%, with individual scores ranging between 0 and 66% (Fig. 2c). Many companies (24/33) committed to reformulation to some extent for at least one nutrient of concern such as sodium (8/26 eligible companies), *trans*-fat (10/30), saturated fat (6/31), added sugars (13/33), or to reduce the energy content or portion size of their products (10/33). Twelve companies were participating in national and/or global industry initiatives on reformulation (e.g. *Healthier Choice Logo* programme or other healthy eating initiatives), whereas no such initiatives were observed in the QSR and retailer sectors. Commitments to product formulation included a lack of nutrient reduction targets, application to only select key products and non-specificity to the Malaysian market. In addition, a company’s self-determination on ‘healthier’ food product composition without external verification affected scores.

Recommendations included to (1) set SMART (specific, measurable, achievable, relevant, and time-bound) national targets for product formulation and regularly report on them; (2) align the targets with *Healthier Choice Logo* and also consider the WHO nutrient profiling systems for all relevant categories. These recommendations were also applicable to the suppliers or third-party manufacturers supplying products to the retailers.

### Nutrition labelling

The overall median score for the nutrition labelling domain was 15% with the highest score recorded as 57% (Fig. 2d). Most of the companies (30/33) disclosed commitments related to nutrition labelling with some companies scoring highest for publishing nutrition information online (16/33) and displaying energy on FOP labelling (17/28). Approximately half of the companies included total/ added sugars (13/28) or *trans*-fat (13/24) content and used *Healthier Choice Logo* or their own FOP formats (14/28) such as numeric information of key nutrients. Less than half of the companies committed to the *Malaysian Food Composition Database* programme (9/33), disclosed some commitments on using nutrition or health claims only for ‘healthy’ products (5/28) or displayed QUIDs labelling (4/28) for their products. Within the QSR sector, nutrition information on request, such as pamphlets on trays or wall charts, was provided by some companies (2/5) but none displayed nutrition information on the menu board. Four retailers participated in a government BeSS (*Clean, Safe and Healthy*) accreditation for food premises, which has a criterion for minimal calorie tagging on menu.

Key recommendations were to (1) provide comprehensive nutrition labelling online and on pack (e.g. sodium, *trans*-fat, sugars) and QUIDs; (2) participate in government-led initiatives such as *Malaysian Food Composition Database* and FOP labelling programmes; and (3) commit that only ‘healthier’ products (*Healthier Choice Logo* and/or WHO criteria) are permitted to carry nutrition claims. For QSR and retailer sectors, nutrition information was recommended to be displayed using the same size fonts as for the price tags on the menu board for all takeaways or ready-to-eat foods prepared on site.

### Promotion practices

The overall median score was zero, with only one-third of companies (11/33) having published some commitments to restrict unhealthy food marketing (Fig. 2e). The limited commitments were to restrict promotion in broadcast and non-broadcast media (10/33), primary schools (9/27) and secondary schools (2/27), limit the usage of celebrity endorsements (7/33), fantasy and animation characters (6/33) and premium offers (5/33), and to undertake policy compliance audits (6/33). Most of the companies with commitments in this area were signatories to the *Malaysia Pledge* and/or other global marketing pledges or policies.

Key recommendations included to (1) establish responsible marketing policies for all media and children’s settings with strict criteria applied to children up to 18 years old, implement time-based restrictions on children’s programming hours, set cut-off at 15–25% or more of children audience viewership, and apply the WHO nutrient profiling systems, and (2) regularly disclose independent national audits of compliance. Retailers were encouraged to promote ‘healthier’ products, in line with WHO criteria, on catalogues, in-store promotion and other activities, and the QSR sector were encouraged to refrain from all forms of advertising in schools including the provision of branded certificates and vouchers.

### Product accessibility

Most companies (21/33) disclosed some commitments on food accessibility but the overall median score was 4% (Fig. 2f). Companies reported commitments to increase availability of some ‘healthier’ products (15/28) and their availability at specific settings (3/28), and general commitments to improve product affordability (10/33). Three companies provided negative statements on taxation as a means to curb intake of unhealthy food products. Lower scores related to the use of “gimmicks through promotional campaigns” (e.g. Buy 1 Free 1) that aimed to drive sales rather than addressing product accessibility, were observed in this study. The QSR sector provided little evidence that they were committed to healthier product accessibility. In some cases, these companies offered mineral water as a value deal or provided ‘healthier’ options on request such as sweetcorn and/or mineral water, with the latter provision incurring additional charges for children’s combination meals.

Recommendations covered the introduction of policies to (1) apply affordable and sustainable pricing (i.e. not short-term ‘price off’ marketing practices) for healthier products compared to non-core products, (2) increase availability through placement strategies of ‘healthier’ products defined by the WHO nutrient profiling systems [42, 43], and (3) support the WHO’s position on fiscal policies [13, 14]. The QSR sector was recommended to (1) introduce ‘healthier’ choices in children and adult combination meals, such as a free water by default or the provision of mineral water, fresh fruits, and vegetable options at no extra charges, and (2) commit to not opening new stores within 500 m of schools.

### Relationships with external organisations

All companies reported at least one type of relationship with external organisations (Fig. 2g) with scores for this domain ranging between 3 and 75%. The CSR activities included funding or in-kind support provided to research (6/30), professional organisations (12/31), nutrition education (12/31) and active lifestyle programmes (20/33), public-private partnerships (15/33), and philanthropic activities (29/33). Some companies explicitly disclosed their position to restrict political donations (9/33). No company made a specific commitment to conduct CSR activities independent of brand, logo or company promotions.

Evidence revealed that companies often reported relationships with external organisations in a non-consolidated manner and/or not specific to the Malaysian market. Therefore, key recommendations included to (1) publicly disclose all national CSR activities in a regular and consolidated manner, and (2) avoid commercial branding and product promotion in their nutrition and healthy lifestyle programmes.

## Discussion

This was the first BIA-Obesity study to assess the nutrition commitments and disclosure practices of major food companies in Asia. In terms of overall scorings for the 33 companies, less than a quarter scored more than 25% (overall median score = 11.0%). Commitments and disclosures of companies in Malaysia were evaluated across six domains of the BIA-Obesity, providing overall median scores for domains ranging between 0 and 28%. Companies performed the worst for commitments related to product formulation, product accessibility, and promotion practices domains (all < 10%). Commitments were often non-specific to the Malaysian market and vague. Across the domains, lower scores were noted related to self-determination of ‘healthy’ products without external verification (e.g. the products that were deemed sufficiently ‘healthy’ to be marketed to children).

The weak nature of food company commitments and disclosures, as discussed above, has also been encountered in other studies. For instance, Cetthakrikul et al. [44] reported food companies in Thailand lacked sufficient specificity and often did not provide detailed criteria in food marketing to children, nutrition and health claims, and food accessibility. A review of commitments and disclosures of companies in 30 countries [33] also revealed wide variations in country specific policies of transnational chain restaurants regarding improving healthfulness of their menus, as well as time commitments to executing these targets. Lack of disclosures and uncertain application of global commitments within Malaysia were identified as issues for most of the global and regional companies. As explained by the ATNF [26], nutrition activities of such companies as reported in their global reports mainly applied to major markets, which might exclude smaller countries such as Malaysia.

The findings from this assessment performed for Malaysia highlights differences in commitments and disclosure practices of companies between high-income countries and low- and middle-income countries (LMICs). As the assessment criteria of BIA-Obesity were modified to suit the Malaysian context, direct country-comparisons could not be reliably made across the board. However, general patterns could still be observed, revealing lower median scores for Malaysia, compared to Australia [28–30], New Zealand [31], and Canada [32]. Moreover, companies that were assessed in both Malaysia and Australia/ New Zealand, typically scored lower in Malaysia. In contrast, companies with market presence in both Malaysia and Canada [32] elicited similar scores in both countries. Sacks et al. [45] suggested that variation of policies within a company for different markets may reflect the different country contexts, including different regulatory pressures, and different consumer demand patterns, but the particular drivers of these variations warrant further exploration.

The *Global Reporting Initiative* (GRI) Standards serve as a guide to companies to practise sustainability reporting to align their corporate position in relation to SDG target 12.6. Publicly listed companies in the Malaysian stock market were more likely than non-listed companies to fulfil GRI as detected by this study, and most of these companies were in line with Malaysia’s NPANM III 2016–2025, the global WHO’s recommendations and/or SDGs. These companies also were participating in Malaysian government-led initiatives such as *Healthier Choice Logo*, BeSS (*Clean, Safe and Healthy*), and/or *Malaysian Food Composition Database* programmes. Additionally, two out of three companies were signatories to the *Malaysia Pledge*, practised an internal marketing policy with compliance audits, and published CSR activities including a policy to restrict political donations. Such activities complement some indicators included in the *Sustainability Reporting Guide* (SRG) recommended by the Bursa Malaysia [46], which is adapted from GRI. Thus, sustainability reporting would likely encourage the inclusion of population nutrition and health strategies into the business model as evidenced by GRI or SRG compliant companies. This study highlights the need for the government to consider regulatory changes to integrate recommendations for areas evaluated by BIA-Obesity Malaysia. This could lead to a nationally endorsed sustainability reporting system related to population nutrition for food and non-alcoholic beverage companies. Furthermore, formulating appropriate fiscal policies (e.g. taxation rebates or incentives) would encourage sustainability reporting and increase transparency in businesses.

Transparency is a key element of accountability [47]. This element was central to recommendations made across most BIA-Obesity domains, which emphasised the importance of public disclosures of company commitments and/or regular reporting for population nutrition and health. Participating companies in our study who provided more evidence for the BIA-Obesity assessment were also more likely to make public their policy information and this concurred with findings from similar studies in New Zealand, Australia, and Canada [28, 31, 32]. These may indicate preparedness for evidence compilation and global company policies tuned towards population nutrition and health. They also may have a greater tendency to publicise related commitments because of global pressure for public-private partnerships to tackle obesity and NCDs prevention [44].

The observed low levels of commitment from companies towards population nutrition puts into question the effectiveness of food industry self-regulation in this area. Moreover, we note that a preference for voluntary industry initiatives in the area of nutrition have been identified as a key strategy used by the food industry as part of efforts to influence public policy in their favour [48]. This ‘policy substitution’ strategy is often coupled with other corporate political activities, such as direct lobbying of government and constituency building activities (including public-private partnerships), to weaken or delay public policy responses [49–51]. In light of well-documented corporate political activities, governments are being urged to implement clear, transparent, and robust guidelines on conflicts of interest and processes to mitigate industry influence on public policy development [23]. Conflict of interest management processes need not completely exclude engagement with industry, particularly as part of policy implementation, but the risks associated with such engagement need to be closely managed. In addition, there is a need for government-led monitoring and evaluation to determine the effectiveness of industry self-regulation on food environment policies.

Varying approaches to nutrition labelling by companies, particularly FOP labelling, were observed in this study. Draper et al. [52] warned that multiple FOP labelling formats in a market would likely limit consumer understanding and lower usage. Relatedly, public health advocates have proposed the application of consistent FOP labelling on all products to improve consumer food choices [53]. Specific to the *Healthier Choice Logo* programme in Malaysia, its use was viewed as conflicting with stronger FOP labelling implementation [54]. A case in point at the time of this study is the *Healthier Choice Logo* criteria were limited to selected foods within categories [55] such as cereals which excluded bread but included instant noodles [55] which is viewed as an ultra-processed food [56].

Few sampled companies in this study had committed to the self-regulatory *Malaysia Pledge* restricting food marketing targeting children. Some concerns regarding the likely population health benefits of the *Malaysia Pledge* were raised at a WHO bi-regional forum [57], pointing to the lack of reliable systems in place to monitor progress, the small number of signatories, and lack of robust nutrition criteria underpinning the pledge. Most of the assessed companies favoured setting age below 12 years as a cut-off to control unhealthy food promotion to children, whereas the United Nations Committee on the Rights of the Child [58] recommended 18 years old as the target cut-off. All these implied the need to strengthen regulation of unhealthy food marketing following strict criteria as per the recommendation of the Review Panel, which was also echoed in an earlier study that called for stronger government-led actions in Malaysia [59].

In the area of product accessibility, a key recommendation from this study emphasised a need for pricing practices that would make healthier products more affordable to the bottom and middle income household groups in Malaysia through sustained lower prices, with less focus on temporary price promotions. A need was also identified for the food retail and manufacturer sectors to improve the placement of healthier products to facilitate easier identification by consumers. The proposed tax incentives for healthier foods as recommended in the NPANM III 2016–2025 [16] may provide incentives for the food industry to shift to the ‘health viable profit’ model, as mooted by Swinburn et al. [23].

In the context of CSR activities, strong branding or product promotion was observed in Malaysia. Since 2007, CSR has been mandatory for companies listed on the Malaysian stock market, with tax incentives for implementation. However, opinion is that most businesses favoured CSR activities linked to philanthropy, rather than population health [46]. Besides, we found little evidence on the effectiveness of reported CSR targeting population health. Kraak et al. [50] indicated that the major challenge in public-private partnerships is to manage conflicts of interest and only allow healthier products for brand-use activities. Therefore, a stricter recommendation was proposed for healthy lifestyle and nutrition programmes to be free from companies’ products and/or brands.

The BIA-Obesity tool assessed the strength of profiling systems used by companies for the purposes of food formulation, labelling, marketing to children, and accessibility. In this study, *WHO nutrient profiling systems* [42, 43] were used as the benchmarks for assessing the healthiness of relevant product portfolios of companies. Recommendations generated by the Review Panel for relevant domains also referred to these profiling systems. The WHO systems enable a country model to consider the regulation of taxation, labelling, and guidelines for healthy food provision in public food service settings [14]. Adoption of the WHO systems would ensure a consistent approach to determining nutrient limits for the classification of a ‘healthy’ product across a range of policy domains [26, 60, 61]. For food companies in Malaysia, it will be important to align relevant policies with ‘reputable’ nutrient profiling systems, rather than generating custom-fit profiling to suit an individual company’s product range.

Through the BIA-Obesity Malaysia process, civil society provided critical assessment and valuable recommendations that focussed on public interests without commercial influence. This academic-led assessment of companies provides strong evidence to the government on the lack of progress in relation to existing self-regulatory policy approaches, which should provide some impetus to shift towards mandatory policies. For the food industry, this study provides evidence of the limitations of existing commitments and disclosure practices to improve related to population nutrition and disclosure practices. A first step for companies would be to formulate SMART commitments and to improve public disclosure of such commitments. The results of this study can also be used by civil society advocates to increase uptake of the study’s recommendations.

This study had several methodological strengths. The tool used has been adapted from INFORMAS, which was earlier implemented in Australia [28–30], New Zealand [31], and Canada [32]. The development process was independent from the food and beverage industry [27]. The tool was modified to suit the local context by taking account of local nutrition policies in the assessment criteria to better reflect a country-specific assessment. The innovation of the process included the appointment of the Expert and Review Panels with balanced representation from the government, NGO and academic backgrounds. A positive characteristic of this study was the test agreement adopting the ‘simple majority rule’ approach which recorded 94.2% agreement between the Expert Panel members. Dissemination of preliminary individual scorecards to companies and holding a closed-door meeting with industry prior to public release of the findings also ensured that the conduct of the study through to the final stages, remained transparent and unbiased.

However, this study had a number of important limitations. Firstly, this study only conducted Phase I of the BIA-Obesity methods, which assessed commitments and disclosures of food companies in relation to population nutrition and health. Although some corporate political activities like corporate philanthropy were assessed to some extent in this study, other practices identified by Mialon et al. [48] such as political lobbying, funding of research and political donations warrant further investigation. A critical evaluation of the healthiness of companies’ product portfolios and the extent of their marketing practices is also recommended as a follow up study. Secondly, the low level of participation by food companies (*n* = 6/33) limited the extent of data collection which led to dependence on only publicly available information for non-participating companies. To overcome issues associated with low participation rates, market surveys were conducted to validate evidence and increase credibility of information presented for assessment. The level of participation from companies likely reflected these companies’ first-time experience with the BIA-Obesity assessment and it is anticipated that future follow-up assessments may overcome this reservation to engage, as has been the experience of similar initiatives elsewhere [25, 26]. If more food companies participated in the BIA-Obesity process, even with the option of providing information on a confidential basis, this is likely to improve the completeness of the evidence, instead of heavily relying on publicly available information. Moreover, future periodic monitoring via BIA-Obesity may improve food company disclosure practices and thus the accuracy of publicly available information used in the assessment.

## Conclusion and policy implications

This study provided an understanding and critical assessment of the Malaysian food industry’s current commitments to improving population nutrition, along with recommendations for change. The lack of efficacious self-regulation in food reformulation, labelling, and marketing, brand-associated CSR activities, and the lack of a uniform credible nutrition profiling system implies that policy makers need to adopt mandatory regulations as part of efforts to create healthier food environments. Appropriate regulatory changes by the government with non-compliance consequences would foster greater adherence of the food industry to policies prioritising population nutrition and health. Furthermore, this study highlighted the need for greater transparency in food company reporting to strengthen accountability for improving population nutrition.

## Supplementary information

**Additional file 1 : Table S1.** Domain Weightings by Sector. Weightings assigned for each domain according to manufacturer, quick service restaurant and retailer sectors.

**Additional file 2 : Table S2.** Indicators for BIA – Obesity Malaysia by Sector. Indicators adapted for BIA-Obesity Malaysia for each domain according to manufacturer, quick service restaurant and retailer sectors.

**Additional file 3 : Table S3.** Market Share of Companies by Food Category. Market share of selected companies by food category according to manufacturer, quick service restaurant and retailer sectors.

**Additional file 4.** Email Template for Industry Engagement. A sample of the email for industry engagement.

**Additional file 5 : Table S4.** Example of an Individual Scorecard. A preliminary scorecard [Ayamas Food Corporation Sdn. Bhd. - a selected company] used in ‘Stage III: Findings finalisation – Feedback of preliminary findings to companies via email’..

## Data Availability

The dataset will be available upon request to the corresponding author, excepting an individual company’s confidential information bound by non-disclosure clauses.
