# Supplementary material for: Benchmarking the transparency, comprehensiveness and specificity of population nutrition commitments of major food companies in Malaysia
Source: Global Health. 2020 Apr 17;16:35. doi: 10.1186/s12992-020-00560-9 (PMC7165366; doi:10.1186/s12992-020-00560-9)
Supplement: Supplementary file 1 — Additional file 1 : Table S1. Domain Weightings by Sector. Weightings assigned for each domain according to manufacturer, quick service restaurant and retailer sectors. [file 12992_2020_560_MOESM1_ESM.docx]

**Table S1 Domain Weightings by Sector**

| Domain |  | Manufacturers | QSR | Retailers |
| --- | --- | --- | --- | --- |
| Corporate strategy | | 10.0 | 10.0 | 10.0 |
| Product formulation | | 30.0 | 25.0 | 25.0 |
| Nutrition labelling | | 20.0 | 15.0 | 15.0 |
| Promotion practices | | 30.0 | 25.0 | 25.0 |
| Product accessibility | | 5.0 | 20.0 | 20.0 |
| Relationships with external organisations | | 5.0 | 5.0 | 5.0 |
| Total | | 100.0 | 100.0 | 100.0 |

*Abbreviation: QSR = quick service restaurants*
