# Supplementary material for: Benchmarking the transparency, comprehensiveness and specificity of population nutrition commitments of major food companies in Malaysia
Source: Global Health. 2020 Apr 17;16:35. doi: 10.1186/s12992-020-00560-9 (PMC7165366; doi:10.1186/s12992-020-00560-9)
Supplement: Supplementary file 2 — Additional file 2 : Table S2. Indicators for BIA – Obesity Malaysia by Sector. Indicators adapted for BIA-Obesity Malaysia for each domain according to manufacturer, quick service restaurant and retailer sectors. [file 12992_2020_560_MOESM2_ESM.docx]

**Table S2 Indicators for BIA – Obesity Malaysia by Sector**

| **Indicator Code** | **Quick Service Restaurants [R]** | **Indicator Code** | **Manufacturers [M]** | **Indicator Code** | **Retailers [S]** |
| --- | --- | --- | --- | --- | --- |
| **Corporate Strategy [STRAT]** | | | | | |
| *Exclusion criteria: Undernutrition or malnutrition (such as nutritional deficiencies); strategies specifically targeting a company’s own employees and contractors, strategies focused on breast milk substitutes and infant formula, nutrient fortification, palm oil and packaging initiatives (related to sustainability).* | | | | | |
| R/ M/ S-STRAT1 | Does the company have an overarching commitment to population nutrition and health articulated in strategic documents (e.g. mission statement, strategies, or overarching policies)? | | | | |
| R/ M/ S-STRAT2 | Does the company’s commitment to population nutrition and health (where it exists) include specific objectives and targets for obesity and NCDs? | | | | |
| R/ M/ S-STRAT2.1 | Contains specific, measurable, achievable, relevant and time bound (SMART) objectives and targets? | | | | |
| R/ M/ S-STRAT2.2 | Recognition or reference to relevant priorities set out in the WHO Global Action Plan for the Prevention and Control of NCDs 2013-2020, Sustainable Development Goals (SDG), or the  WHO Report on Ending Childhood Obesity? | | | | |
| R/ M/ S-STRAT2.3 | Recognition or reference to relevant priorities in national government policy documents relating to population nutrition and obesity/ NCD prevention? | | | | |
| R/ M/ S-STRAT2.4 | Comprehensive in nature (e.g. includes three or more domains in this document, such as formulation, marketing and labelling)? | | | | |
| R/ M/ S-STRAT2.5 | Key Performance Indicators (KPIs) (and/or remuneration) of senior managers linked to nutrition strategy/ policy/ targets? | | | | |
| R/ M/ S-STRAT3 | Does the company regularly publish details of its approach to population nutrition and health related to obesity and NCDs? | | | | |
|  |  |  |  |  |  |
| **Product Formulation [FORM]** | | | | | |
| *Exclusion criteria: Reformulation/ product development relating to other macronutrients or micronutrients, nutrient fortification, allergens, and palm oil initiatives.* | | | | | |
| R/ M/ S-FORM1 | Does the company publish a comprehensive set of commitments or objectives related to new product development and reformulating its existing products (or [R]: in-house products) with respect to reducing the nutrients of concern and energy (salt, saturated fats, *trans*-fats, added sugars and calorie)? | | | | |
| R/ M/ S-FORM2 | Is the company a signatory to national^a^ and/or global industry initiatives on product reformulation or do they commit or participate to national voluntary government programmes on product (or [R]: in-house product) reformulation (where applicable)? | | | | |
| R/ M/ S-FORM3.1 | Has the company set a target/ targets or taken substantive action to reduce/ reach lower levels of salt/ sodium in products (or [R]: for in-house products)? | | | | |
| R/ M/ S-FORM4.1 | Has the company set a target/ targets or taken substantive action to reduce artificial *trans*-fat added to products (or [R]: in-house products) during the manufacturing process? | | | | |
| R/ M/ S-FORM5.1 | Has the company set a target/ targets or taken substantive action to reduce/ reach lower levels of saturated fats (or [R]: for in-house products)? | | | | |
| R/ M/ S-FORM6.1 | Has the company set a target/ targets or taken substantive action to reduce/ reach lower levels of added sugars (or [R]: for in-house products)? | | | | |
| R/ M/ S-FORM7.1 | Does the company have a target/ targets or taken substantive action to reduce the portion size/ energy content of single serve snacks (or [R]: for in-house products)? | | | | |
| R/ M/ S-FORM8.1 | What system/ criteria (e.g. product classification system or nutrient profiling system) does the company use for the purposes of product (or [R]: in-house brand or “private label” products) development/ reformulation? | | | | |
| R/ M/ S-FORM9.1 | Does the company publish its policy position (in relation to government policy, where government policy exists^a^) on product (or [R]: in-house brand or “private label” product) reformulation? | | | | |
| R/ M/ S-FORM9.2 | Does the company’s policy position^a^ support WHO’s position on product (or [R]: in-house product) reformulation in relation to nutrients of concern (e.g. salt, added sugar, saturated fat and *trans*-fat), as articulated in the Global Action Plan for the Prevention and Control of NCDs 2013-2020? | | | | |
|  | | | | | |
| **Nutrition Labelling [LABEL]** | | | | | |
| *Exclusion criteria: Government mandated nutrition information or product labelling information (e.g. total sugars declaration on ready-to-drink products), allergen information, criteria for the nutrition claims mandated by Food Regulations 1985, and sustainable packaging initiatives.* | | | | | |
| R-LABEL1 | Does the company commit to disclose nutrition information on its menus (i.e. menu board labelling)? | M/ S-LABEL1 | Does the company commit to disclose quantitative ingredient declarations (QUIDs) on its products (or [R]: in-house products)? | | |
| R-LABEL2.1 | What nutrition information does the company commit to provide on menus (i.e. menu boarding labelling – calories, nutrients of concern, symbol and etc.)? | M/ S-LABEL2.1 | Does the company commit to disclose nutrition information on its products (or [R]: in-house brand products) on ‘per 100g/100mL’ basis? | | |
| R-LABEL2.2 | Is the nutrition information on menus (i.e. menu board labelling) presented in the same size and font as price? | M/ S-LABEL2.3 | Does the company commit to provide on-pack information on *trans*-fat (TFA) content (or [R]: for own-brand products)? | | |
| R-LABEL2.3 | If energy/ calorie information is displayed on menus (e.g. menu board labelling), does the company provide a contextual statement regarding the number of kilojoules (kJ)/ calories that should be consumed in a day for the average adult to maintain a healthy weight? | M/ S-LABEL2.4 | Does the company commit to provide on-pack information on added sugars and/or total sugars content (or [R]: for in-house brand products)? [Note: For beverage company e.g. soft-drink and/or juice portfolio, the scoring scheme will assess ‘added sugar’ labelling only, but not ‘total sugar’ labelling as it is mandatory to label in ready-to-drink beverages according to Food Regulations 1985.]. | | |
| R-LABEL3 | Does the company provide nutrition information upon request on-site? | M/ S-LABEL5.1 | Does the company have a published commitment to rolling out the government-endorsed front-of-pack (FOP) labelling (i.e. a single icon for ‘energy’ based on a daily calorie intake of 2000kcal of a normal adult) scheme (or [R]: on in-house brand products)? | | |
| R-LABEL4/  M/ S-LABEL3 | Does the company provide information on food composition (or [R]: of in-house brand products) to national authorities, on request? | | | | |
| R-LABEL5/ M/ S-LABEL4 | Does the company provide nutrition information online (or [R]: for in-house brand products and others)? | | | | |
| R-LABEL6 | Does the company publish its policy position (in relation to government policy or others) on menu labelling? | M/ S-LABEL5.2 | Does the company commit to use a ‘comprehensive’ FOP labelling system (or [R]: on in-house brand products)? (Note: Beyond a single icon for “energy” FOP e.g. traffic lights, warning labels, Health Star Rating (HSR) and others^a^, but exclude back-of-pack labelling) | | |
|  | | M/ S-LABEL5.3 | What system/ criteria (e.g. product classification system or nutrient profiling system^a^) does the company use to classify the healthiness of products for the purposes of FOP nutrition labelling (or [R]: for in-house brand products)? | | |
|  |  | M/ S-LABEL6.1 | Does the company publish its policy position (in relation to government policy) on FOP labelling (or [R]: for in-house brand products)? | | |
|  |  | M/ S-LABEL6.2 | Does the company’s policy position^a^ support WHO’s position on FOP nutrition labelling (or [R]: for in-house brand products), as articulated in the WHO Report? | | |
|  |  | M/ S-LABEL7^b^ | Does the company state that it will place a nutrient function claim (i.e. one of the health claims as per CODEX’s definition e.g. Beta-glucan helps to reduce cholesterol) on a product (or [R]: an in-house brand product) (or use a health claim as part of product advertising) only when the product is 'healthy', and in compliance with conditions in CODEX? | | |
|  |  | M/ S-LABEL8^b^ | Does the company state that it will place a nutrition claim on a product (or [R]: an in-house product) (or use a nutrition claim as part of product advertising) only when the product is 'healthy'? | | |
|  |  | M/ S-LABEL9.1 | What system/ criteria (e.g. product classification system or nutrient profiling system) does the company use to classify the healthiness of products (or [R]: in-house brand products) for the purposes of health and/or nutrition claims? | | |
|  |  |  | | S-LABEL10 | Does the company use shelf tags that provide summary nutrition information (e.g. Guiding Stars, HSR, NuVal)? |
|  |  |  |  | S-LABEL11 | Does the company have an ongoing nutrition/ healthy eating education programme in-store? (e.g. dietitians in stores, nutrition education materials, etc.) |
|  |  |  |  | S-LABEL12.1 | Does the company commit to disclose nutrition information (e.g. on menus) for takeaway or ready-to-eat (RTE) foods prepared on site? |
|  |  |  |  | S-LABEL12.2 | What nutrition information does the company commit to provide (e.g. on menus – calories, nutrients of concerns, symbol, and etc.) for takeaway or RTE foods prepared on site? |
|  |  |  |  | S-LABEL12.3 | Is the nutrition information for takeaway or RTE foods prepared on site presented in the same size and font as price? |
|  |  |  |  | S-LABEL12.4 | If energy/ calorie information is displayed, does the company provide a contextual statement regarding the number of kJ/ calories that should be consumed in a day for the average adult to maintain a healthy weight? |
|  |  |  |  | S-LABEL12.5 | Does the company provide nutrition information for takeaway or RTE foods that are prepared on site upon request on-site? |
|  |  |  |  | S-LABEL13 | Does the company publish its policy position (in relation to government policy, if it exists) on menu labelling (i.e. for takeaway or ready-to-eat foods that are prepared on site)? |
|  | | | | | |
| **Promotion Practices [PROMO]** | | | | | |
| *Exclusion criteria: Promotion to adults over 18 years old (except ret*ailer sector), other aspects of marketing such as price or place, advertising of breast milk substitutes. | | | | | |
| R/ M/ S-PROMO1.1 | Does the company have a policy^c^ to reduce the exposure of non-core food marketing to children on broadcast media (TV, radio)? | | | | |
| R/ M/ S-PROMO1.2 | To what age group(s) does the broadcast marketing policy apply? | | | | |
| R/ M/ S-PROMO1.3 | How is the ‘target audience’ or ‘audience exposed’ defined? | | | | |
| R/ M/ S-PROMO2.1 | Does the company have a policy^c^ to reduce the exposure of non-core food marketing to children on non-broadcast media (including websites, DVDs/ games, social media, print media, product placement, outdoor marketing, and/or in store marketing/ point of sales marketing)? | | | | |
| R/ M/ S-PROMO2.2 | To what age group(s) does the non-broadcast marketing policy apply? | | | | |
| R/ M-PROMO3 | Does the company commit not to sponsor children’s sporting, cultural or other activities using non-core brands (foods or company brands)? | | | S-PROMO7 | Does the company have a marketing policy to reduce exposure of all consumers to non-core food marketing? |
| R/ M-PROMO4 | Does the company commit not to use marketing in settings where children gather using non-core brands (foods or company brands)?^c^ | | | S-PROMO8 | To which media/ settings does the marketing policy (related to all consumers) apply? |
| R/ M-PROMO4.1 | Commits IN early childcare settings and primary schools (e.g. children up to age 12)? | | | S-PROMO8.1 | Broadcast media (TV, radio)? |
| R/ M-PROMO4.2 | Commits NEAR (e.g. within 500m) of early childcare settings and primary schools (e.g. children up to age 12)? | | | S-PROMO8.2 | Non-broadcast media (including  websites, DVDs/ games, social  media, print media, product  placement, outdoor marketing)? |
| R/ M-PROMO4.3 | Commits IN secondary schools (e.g. children between age 13 and 18)? | | | S-PROMO9 | Does the company have a policy to limit their in-store promotion of non-core products? |
| R/ M-PROMO4.4 | Commits NEAR (e.g. within 500m) of secondary schools (e.g. children between age 13 and 18)? | | | S-PROMO10 | Does the company have a policy on the proportion of healthy (compared with non-core foods) foods promoted in their regular catalogues/ circulars? |
| R/ M-PROMO4.5 | Commits in other places where children gather (e.g. family and child clinics, paediatric services or other health facilities, sporting or recreation centres, and/or sporting or cultural events held at those premises)? | | | S-PROMO11 | Does the company have a policy to link rewards programmes or loyalty programmes to healthy food items? |
| R/ M-PROMO5.1/ S-PROMO3.1 | Does the company pledge not to use celebrities in marketing of products to children other than those that meet the company’s ‘healthy’ standard? | | | | |
| R/ M-PROMO5.2/ S-PROMO3.2 | Does the company pledge not to use fantasy and animation characters with a strong appeal to children in marketing of products other than those that meet the company’s ‘healthy’ standard? | | | | |
| R/ M-PROMO5.3/ S-PROMO3.3 | Does the company commit not to use premium offers (e.g. promotional toys, games, vouchers and competitions) in marketing of products other than those that meet the company’s ‘healthy’ standard? | | | | |
| R-PROMO6 | Does the company commit to only advertise or display ‘healthy’ sides and ‘healthy’ drinks in children’s combination meals in restaurants (for example, on menus and menu boards; or in advertisements in restaurants)? |  | | S-PROMO12 | Does the company have a policy to ensure that in-store product presentations, product giveaways or tastings are for healthy products (including giveaways to children)? |
| R-PROMO7/  M-PROMO6/  S-PROMO4 | Does the company audit its compliance with its policy on marketing to children at the national/ country level? | | | | |
| R-PROMO8.1/ M-PROMO7.1/  S-PROMO5.1 | What system/ criteria (e.g. product classification system or nutrient profiling system) does the company use to classify the healthiness of products for the purposes of promotion to children? | | | | |
| R-PROMO9.1/ M-PROMO8.1/ S-PROMO6.1 | Does the company publish its policy position (in relation to government policy e.g. the *Malaysia Pledge* 2013, Guideline on the Advertising and Nutrition Information Labelling of Food Foods, etc. on own/ industry association website) on reducing the exposure of children and/or adolescents to, and the power of, the marketing of non-core foods? | | | | |
| R-PROMO9.2/  M-PROMO8.2/ S-PROMO6.2 | Does the company’s policy position support WHO’s position on government-led policy action related to reducing the exposure of children and adolescents to, and the power of, the marketing of non-core foods, as articulated in the WHO Global Action Plan for NCDs and other key WHO documents (such as the *Report of the Commission on Ending Childhood Obesity*)? | | | | |
|  | | | | S-PROMO13 | Does the company audit its compliance with its policy on marketing to all consumers at the national/ country level? |
|  |  |  |  | S-PROMO14.1 | What system/ criteria (e.g. product classification system or nutrient profiling system) does the company use to classify the healthiness of products for the purposes of product promotion to all consumers (e.g. in-store catalogues, brochures, flyers, shelf tags, promotional posters)? |
|  |  |  |  |  |  |
| **Product Accessibility [ACCESS]** | | | | | |
| *Exclusion criteria: Decreasing/ increasing the price of products due to supply and demand.* | | | | | |
| R-ACCESS1 | Does the company make a commitment to address the price/ affordability of healthy products relative to its non-core products? | M-ACCESS1 | Does the company make a commitment to address the price/ affordability of its healthier products relative to its non-core products (e.g. the company manufactures both ‘healthy’ and ‘non-core’ products)? | S-ACCESS1 | Does the company make a commitment to address the price/ affordability of healthy products relative to its non-core products, particularly where there are comparable substitutes (e.g. lower/ equivalent standard price)? |
| R-ACCESS2 | Does the company have a policy that price promotions and ‘value deals’ are used only on healthy products? | M-ACCESS2/ S-ACCESS5.1 | Does the company have a policy to increase the number/ proportion of healthy products in the company’s portfolio? | | |
| R-ACCESS3 | Does the company commit to not use price incentives (such as ‘supersizing’) that incentivise consumers to purchase larger portion sizes for minimal extra cost? | M-ACCESS3/ S-ACCESS5.2 | Does the company make a clear and specific commitment to increase the availability of healthy products and/or decrease the availability of non-core products in specific settings? | | |
| R-ACCESS9.1/ M-ACCESS4.1/ S-ACCESS10.1 | What system/ criteria (e.g. product classification system or nutrient profiling system) does the company use to classify the healthiness of products for the purposes of product pricing, distributing and/or availability (or [R]: (e.g. dedicated amount of shelf space, product placement at end of aisles/ high traffic areas, product placement at checkout)? | | | | |
| R-ACCESS10.1/ M-ACCESS5.1/ S-ACCESS4.1 | Does the company publish its policy position (in relation to government policy, where it exists) on fiscal policies to make healthier foods relatively cheaper and non-core foods relatively more expensive? | | | | |
| R-ACCESS10.2/ M-ACCESS5.2/ S-ACCESS4.2 | Does the company’s policy position support WHO’s position on fiscal policies to make healthier foods relatively cheaper and non-core foods relatively more expensive, as articulated in the *WHO Global Action Plan for NCDs* and the *Report of the Commission on Ending Childhood Obesity*? | | | | |
| R-ACCESS4 | Does the company commit to not provide free refills for caloric soft drinks/ soda? |  | | S-ACCESS2 | Does the company have a published position on the size and nature of discounts/ price promotions applied to healthy and non-core foods (e.g. no discount for non-core, greater discount for healthy foods across food categories, etc.)? |
| R-ACCESS5 | Does the company commit to not opening new stores near schools? |  |  | S-ACCESS3 | Does the company make a commitment to limit multi-buy specials (e.g. two for one) on non-core foods? |
| R-ACCESS6.1 | Does the company have a policy that ‘assigned’ or ‘default’ drink items (included as part of adult’s combination meals) are healthy items (e.g. water)? |  |  | S-ACCESS6 | Does the company make a clear and specific commitment to dedicate a minimum amount or proportion of shelf space or floor space to healthy products? |
| R-ACCESS6.2 | Does the company have a policy that ‘assigned’ or ‘default’ drink items (included as part of children’s combination meals) are healthy items (e.g. water)? |  |  | S-ACCESS7 | Does the company make a clear and specific commitment to dedicate a maximum amount or proportion of shelf space or floor space to non-core products? |
| R-ACCESS7.1 | Does the company have a policy that ‘assigned’ or ‘default’ side items (included as part of adult’s combination meals) are healthy items (e.g. salad, vegetables)? |  |  | S-ACCESS8 | Does the company have a policy that checkouts are free from non-core items (including confectionery, chocolate and soft drinks)? |
| R-ACCESS7.2 | Does the company have a policy that ‘assigned’ or ‘default’ side items (included as part of children’s combination meals) are healthy items (e.g. salad, vegetables)? |  |  | S-ACCESS9 | Does the company have a published position on the placement of non-core items (such as confectionery, chocolate and soft drinks) at end of aisle displays or other high-traffic areas? |
|  | | | | | |
| **Relationships with External Organisations [RELAT]** | | | | | |
| *Exclusion criteria: Activities conducted for the suppliers, distributors, retailers, employees, contractors, and service providers.* | | | | | |
| R/ M/ S-RELAT1 | Does the company publish details of the professional organisations (e.g. professional associations for nutrition or dietetics, physical activity or exercise organisations, medical organisations or societies, etc.) and/or scientific events (e.g. conferences) it funds or supports, including awards/ prizes, making clear the nature of that support? | | | | |
| R/ M/ S-RELAT2 | Does the company publish details of the external research (e.g. conducted by individuals/ groups/ organisations) it funds or supports, including awards/ prizes? | | | | |
| R/ M/ S-RELAT3 | For philanthropic funding, does the company publish details of the groups or organisations it funds or supports? | | | | |
| R/ M/ S-RELAT4.1 | Does the company publish details of the nutrition education/ healthy diet-oriented programmes it funds or supports? | | | | |
| R/ M/ S-RELAT5 | Does the company publish details of the active lifestyle programmes (sports, physical activity) it funds or supports? | | | | |
| R/ M/ S-RELAT6 | Does the company publish details of its involvement in public-private partnerships and/or joint ventures with government organisations/ agencies? (Note: In addition to those covered as part of RELAT4.1 and RELAT5. e.g. recycling, infrastructure support – computer labs) | | | | |
| R/ M/ S-RELAT7 | Does the company publish details of its political donations? If yes, please indicate. | | | | |
| R/ M/ S-RELAT8 | Does the company publish its membership/ support for/ ownership of industry associations, think tanks, interest groups, community organisations or other organisations (e.g. membership in Federation of Malaysian Manufacturers) that lobby in relation to population nutrition and/or obesity and NCD issues? | | | | |

*Abbreviations: DVDs=Digital versatile discs; FOP=Front-of-pack labelling; HSR=Health Star Rating; kJ=kilojoules; KPIs=Key Performance Indicators; NCDs=Non-communicable diseases; QUIDs=Quantitative ingredient declarations; RTE-Ready-to-eat; SDG=Sustainable Development Goals; SMART=Specific, measurable, achievable, relevant, time bound; TFA=trans-fat; TV=Television; WHO=World Health Organisation.*

^a^Includes voluntary adaptation of the Malaysian *Healthier Choice Logo* (HCL) as reformulation and labelling efforts.

^b^Includes voluntary industry policy – the *Malaysia Pledge* for restricting unhealth food marketing to children.

^c^Specifies policies related to nutrient function claims and nutrition claim in accordance to permitted claims in the Malaysia Food Regulations 1985.

Notes:

1. Non-core foods are defined as products high in undesirable nutrients, such as total or saturated/ *trans*- fat, added/ free sugars, and sodium/ salt not meeting specific nutrition criteria.
2. M/ S-LABEL 2.2 indicator - “What nutrition information does the company commit to providing (Note: "on in-house brand products" for retailer sector)?” does not apply in this assessment (information only).
3. For M/ S-LABEL7 indicator, ‘…conditions in CODEX’ refer to essential nutrients for which a Nutrient Reference Value (NRV) has been established. Food, for which the claim is made should be a significant source of the nutrient in the diet and based on scientific consensus. A claim should not include any statement that the nutrient would afford a cure or treatment for or protection from a disease.
4. For the retailer sector, indicators with “[R]” refer to in-house brand products.
