# Supplementary material for: Benchmarking the transparency, comprehensiveness and specificity of population nutrition commitments of major food companies in Malaysia
Source: Global Health. 2020 Apr 17;16:35. doi: 10.1186/s12992-020-00560-9 (PMC7165366; doi:10.1186/s12992-020-00560-9)
Supplement: Supplementary file 3 — Additional file 3 : Table S3. Market Share of Companies by Food Category. Market share of selected companies by food category according to manufacturer, quick service restaurant and retailer sectors. [file 12992_2020_560_MOESM3_ESM.docx]

**Table S3 Market Share of Companies by Food Category**

1. **Food and beverage manufacturer sector**

| **National Food and Beverage Company Name** | **Mean of % Retail Value RSP** | | | % Retail Value RSP | | | | | | | | | | | | | | | | | | | | | |
| --- | --- | --- | --- | --- | --- | --- | --- | --- | --- | --- | --- | --- | --- | --- | --- | --- | --- | --- | --- | --- | --- | --- | --- | --- | --- |
|  |  |  |  | Main Category as per Euromonitor Definition^a^ | | | | | | | | | Sub-category as per Euromonitor Definition^a^ | | | | | | | | | | | | |
|  | **Overall** | **Food** | **Beverage** | Baked Goods | Biscuits | Confectionery | Ice-cream | Ready Meals | Savoury Snacks | Processed Meat & Seafood | Soup | Spread | RTE Cereal | Instant Noodles | Cheese | Drinking Milk Products | Yoghurt Products | Other Dairy | Carbonates | Concentrates | Juice | RTD Coffee | RTD Tea | Sports and Energy Drinks | Asian Specialty Drinks |
| 1. Fraser & Neave Holdings Bhd | 11.3 | 5.9 | 22.9 |  |  |  | 16.4 |  |  |  |  |  |  |  |  | 9.2 |  | 62.9 | 28.8 | 6.3 | 15.9 |  | 28.2 | 61.0 | 20.1 |
| 2. Nestlé (M) Bhd | 10.9 | 11.2 | 10.2 |  |  | 15.9 | 23.5 |  |  |  |  |  | 39.4 | 48.4 |  | 13.2 | 24.0 | 3.1 |  |  |  | 71.3 |  |  |  |
| 3. Yeo Hiap Seng (M) Bhd | 6.0 | 5.1 | 8.0 |  |  |  |  | 46.5 |  | 0.6 |  | 2.5 |  | 12.3 |  | 13.9 |  |  |  |  | 4.4 |  | 25.3 |  | 26.6 |
| 4. Mondelēz (M) Sales Sdn Bhd | 4.0 | 5.9 |  |  | 13.2 | 14.9 |  |  | 15.0 |  |  |  |  |  | 45.6 |  |  |  |  |  |  |  |  |  |  |
| 5. Etika Group of Companies^b^ | 3.7 | 1.1 | 9.3 |  |  |  |  |  |  |  |  |  |  |  |  |  |  | 16.7 | 16.7 |  | 15.6 | 18.8 | 4.4 | 9.3 |  |
| 6. Campbell Soup SEA Sdn Bhd | 3.4 | 4.9 |  |  | 0.9 |  |  |  |  |  | 72.8 |  |  |  |  |  |  |  |  |  |  |  |  |  |  |
| 7. Malaysia Milk Sdn Bhd | 3.3 | 3.2 | 3.6 |  |  |  |  |  |  |  |  |  |  |  |  | 8.5 | 34.5 | 4.5 |  |  | 22.8 |  | 2.7 |  |  |
| 8. Unilever (M) Holdings Sdn Bhd | 3.2 | 4.7 |  |  |  |  | 26.2 | 7.9 |  |  | 10.1 | 25.7 |  |  |  |  |  |  |  |  |  |  |  |  |  |
| 9. Coca-Cola Malaysia | 3.1 |  | 9.8 |  |  |  |  |  |  |  |  |  |  |  |  |  |  |  | 46.9 |  | 4.7 |  | 16.7 |  |  |
| 10. Fonterra Brands (M) Sdn Bhd | 2.2 | 3.2 |  |  |  |  |  |  |  |  |  |  |  |  | 29.3 | 16.1 | 2.6 | 0.2 |  |  |  |  |  |  |  |
| 11. Kellogg Asia Marketing Inc | 2.2 | 3.2 |  |  |  |  |  |  | 6.6 |  |  |  | 41.4 |  |  |  |  |  |  |  |  |  |  |  |  |
| 12. Barkath Co-Ro Mfg Sdn Bhd | 1.6 |  | 5.0 |  |  |  |  |  |  |  |  |  |  |  |  |  |  |  |  | 35.0 |  |  |  |  |  |
| 13. Dutch Lady Milk Industries Bhd | 1.5 | 2.2 |  |  |  |  |  |  |  |  |  |  |  |  |  | 23.3 | 9.7 |  |  |  |  |  |  |  |  |
| 14. Mamee-Double Decker (M) Sdn Bhd | 1.4 | 2.0 |  |  |  |  |  |  | 10.3 |  |  |  |  | 15.1 |  |  | 4.5 |  |  |  |  |  |  |  |  |
| 15. Gardenia Bakery KL Sdn Bhd | 1.2 | 1.7 |  | 19.8 |  |  |  |  |  |  |  | 5.8 |  |  |  |  |  |  |  |  |  |  |  |  |  |
| 16. Hup Seng Perusahaan Makanan (M)  Sdn Bhd | 0.8 | 1.2 |  |  | 11.5 |  |  |  | 6.5 |  |  |  |  |  |  |  |  |  |  |  |  |  |  |  |  |
| 17. Munchy Food Industries Sdn Bhd | 0.8 | 1.1 |  |  | 12.1 |  |  |  | 4.7 |  |  |  |  |  |  |  |  |  |  |  |  |  |  |  |  |
| 18. Ferrero SpA | 0.6 | 0.9 |  |  |  | 9.6 |  |  |  |  |  | 4.5 |  |  |  |  |  |  |  |  |  |  |  |  |  |
| 19. Clouet & Co (KL) Sdn Bhd | 0.6 | 0.9 |  |  |  |  |  |  |  | 13.1 |  |  |  |  |  |  |  |  |  |  | 1.1 |  |  |  |  |
| 20. The Italian Baker Sdn Bhd | 0.6 | 0.8 |  | 12.2 |  |  |  |  |  |  |  |  |  |  |  |  |  |  |  |  |  |  |  |  |  |
| 21. Ayamas Food Corp Sdn Bhd | 0.3 | 0.5 |  |  |  |  |  |  |  | 7.4 |  |  |  |  |  |  |  |  |  |  |  |  |  |  |  |
| 22. Ramly Food Processing Sdn Bhd | 0.3 | 0.5 |  |  |  |  |  |  |  | 7.4 |  |  |  |  |  |  |  |  |  |  |  |  |  |  |  |
| **Total % Retail Value RSP** | 62.9 | 60.1 | 68.9 | 32.0 | 37.7 | 40.4 | 66.1 | 54.4 | 43.1 | 28.5 | 82.9 | 38.5 | 80.8 | 75.8 | 74.9 | 84.2 | 75.3 | 87.4 | 92.4 | 41.3 | 64.5 | 90.1 | 77.3 | 70.3 | 46.7 |

|  | RSP values not ranked as top in sequence but contributed significant proportion to market share, according to the corresponding category or sub-category. |
| --- | --- |

*Abbreviations: RSP = retail selling price; RTD = Ready-to-drink; RTE = Ready-to-eat.*

*Notes:*

^a^’Packaged Foods’ and ‘Soft Drink’ categories under Euromonitor International were referred to derive the selection of main and sub-categories.

^b^Etika Group of Companies included Etika Beverages Sdn. Bhd. (manufacturer for soft drinks) and Etika Dairies Sdn. Bhd. (manufacturer of other dairy namely condensed or evaporated milk).

Retail value was derived from RSP track monetary value of packaged food or soft drink sales through retail channels (but excluded hotels, restaurant, cafes, duty free sales and institutional sales namely hospitals, school or work canteens) using retail selling prices (RSP). RSP included the impact of wholesaler, distributor or retailer mark-ups and value-added tax on the item’s price, and reflected the price the consumer pays for the product in the store. Total % Retail Value RSP was rounded to one decimal place.

Source: Datasets Extracted from Euromonitor International [34-36]

**(b) Food and beverage manufacturer sector**

| **National Company Name**  **(Common/ Brand Name)** | Mean of % Foodservice RSP value | % Foodservice RSP value | |
| --- | --- | --- | --- |
|  |  | Fast Food | Pizza Consumer Foodservice |
| 1. QSR Stores Sdn Bhd   (Pizza Hut) | 26.3 |  | 52.5 |
| 2. QSR Stores Sdn Bhd  (KFC) | 21.0 | 41.9 |  |
| 1. Dommal Food Services Sdn Bhd   (Domino’s) | 15.8 |  | 31.5 |
| 4. Gerbang Alaf Restaurants Sdn Bhd  (McDonald’s) | 13.8 | 27.5 |  |
| 5. Golden Donuts Sdn Bhd  (Dunkin’ Donuts) | 2.4 | 4.8 |  |
| **Total % Foodservice Value RSP** | 79.1 | 74.2 | 84.0 |

*Abbreviation: RSP = retail selling price*

Notes:

1. Foodservice RSP value refers to system-wide sales through all units or outlets that were either company owned or managed, franchised or licensed. However, it excluded franchise royalty fees and other payments. Euromonitor’s “Consumer Foodservice” data measured value as the total price paid by consumers including value-added tax at foodservice outlets in all non-captive foodservice categories (e.g. fast food, 100% home delivery or takeaway, etc.). Total % Foodservice Value RSP was rounded to one decimal place.

Source: Dataset Extracted from Euromonitor International [37]

**(C) Retailer sector**

| **National Company Name**  **(Common/ Brand Name)** | Mean of % Retail Value RSP |
| --- | --- |
| 1. GCH Retail (M) Sdn Bhd | 9.3 |
| 2. Tesco Stores (M) Sdn Bhd | 7.0 |
| 3. 7-Eleven Malaysia Sdn Bhd | 3.4 |
| 4. AEON Group | 2.9 |
| 5. Econsave Cash & Carry Sdn Bhd | 2.6 |
| 6. Mydin Mohamed Holdings Bhd | 1.0 |
| **Total % Retail Value RSP** | 26.2 |

*Abbreviation: RSP = retail selling price*

Notes:

1. No food category for retailer sector. Hence, RSP value in retailer sector was referred. The RSP value excluded sales tax, which measured the value of sales generated from retailing activities but excluded sales tax. Total % Retail Value RSP was rounded to one decimal place.
2. AEON Group included AEON Big (M) Sdn Bhd and AEON Co (M) Bhd.
3. Mydin Mohamed Holdings Bhd included Mydin and Kedai Rakyat 1Malaysia outlets in Year 2016.

Source: Datasets Extracted from Euromonitor International [38]
