# Supplementary material for: Benchmarking the transparency, comprehensiveness and specificity of population nutrition commitments of major food companies in Malaysia
Source: Global Health. 2020 Apr 17;16:35. doi: 10.1186/s12992-020-00560-9 (PMC7165366; doi:10.1186/s12992-020-00560-9)
Supplement: Supplementary file 4 — Additional file 4. Email Template for Industry Engagement. A sample of the email for industry engagement. [file 12992_2020_560_MOESM4_ESM.docx]

Additional File 4 Email Template for Industry Engagement

Dear Madam / Mr. ______,

Greetings from UKM! I am William, a research fellow for Prof T Karupaiah and the Business Impact Assessment (BIA) - Obesity Project. Our research team is conducting the first Malaysian private sector assessment related to food environment and nutrition policies for obesity and non-communicable diseases prevention.

[2] We are working with Federation of Malaysian Manufacturers (FMM) to contact members to conduct this survey. Some FMM members might have attended a preliminary project briefing session held at FMM on 8 Nov 2017. FMM is facilitating this engagement process and suggested you, on behalf of your company as the potential candidate to approach for this project.

[3] We acknowledge that this engagement process might require internal approval from top management. Your support will be the first step to facilitate a better communication. To explain this project, we are providing:

i. A cover letter

ii. Research Information Sheet

iii. Brochure for BIA-Obesity

iv. A consent form**

*Note: FYI, the project has been reviewed and approved by Economic Planning Unit (EPU), Prime Minister’s Department (Please refer to the attached file for more details). It is really important for us to be able to liaise with a company representative – otherwise our analysis for this project will be limited to publicly available information (online).*

[4]   As we plan to commence the data verification in ______ 2018, we would appreciate if Company Name can provide feedback **before**  Date . Please fill up the consent form (Document IV)** to indicate your participation.

If you need additional clarification for this project, please do not hesitate to contact me via my mobile number +6016 H/P number or email ( [______@gmail.com](mailto:______@gmail.com) ). We are happy to have a face-to-face discussion at a convenient time and venue to address your doubts too. Lastly, we appreciate your attention, and we look forward to hearing from you.

*Remarks:*

*i. We will appreciate if you can acknowledge receipt of this email.*

*ii. We will mail out the attached files as per the address in the cover letter. Please suggest the corresponding mailing address and suggested person-in-charge to be contacted, if you do not receive it next week. We will respond to you ASAP.*

​

[**Appendix BIA-Obesity**](https://drive.google.com/drive/folders/17z6upI_qSzblPeTJjza9uVHDDgKp3fCG)

​

Yours sincerely,

William Ng

Research Fellow of BIA-Obesity

(on behalf of Prof. Tilakavati Karupaiah – Project Leader BIA-Obesity)
