# Supplementary figures and images for: Benchmarking the transparency, comprehensiveness and specificity of population nutrition commitments of major food companies in Malaysia
Source: Global Health. 2020 Apr 17;16:35. doi: 10.1186/s12992-020-00560-9 (PMC7165366; doi:10.1186/s12992-020-00560-9)

**Table S4 Example of an Individual Scorecard**

**
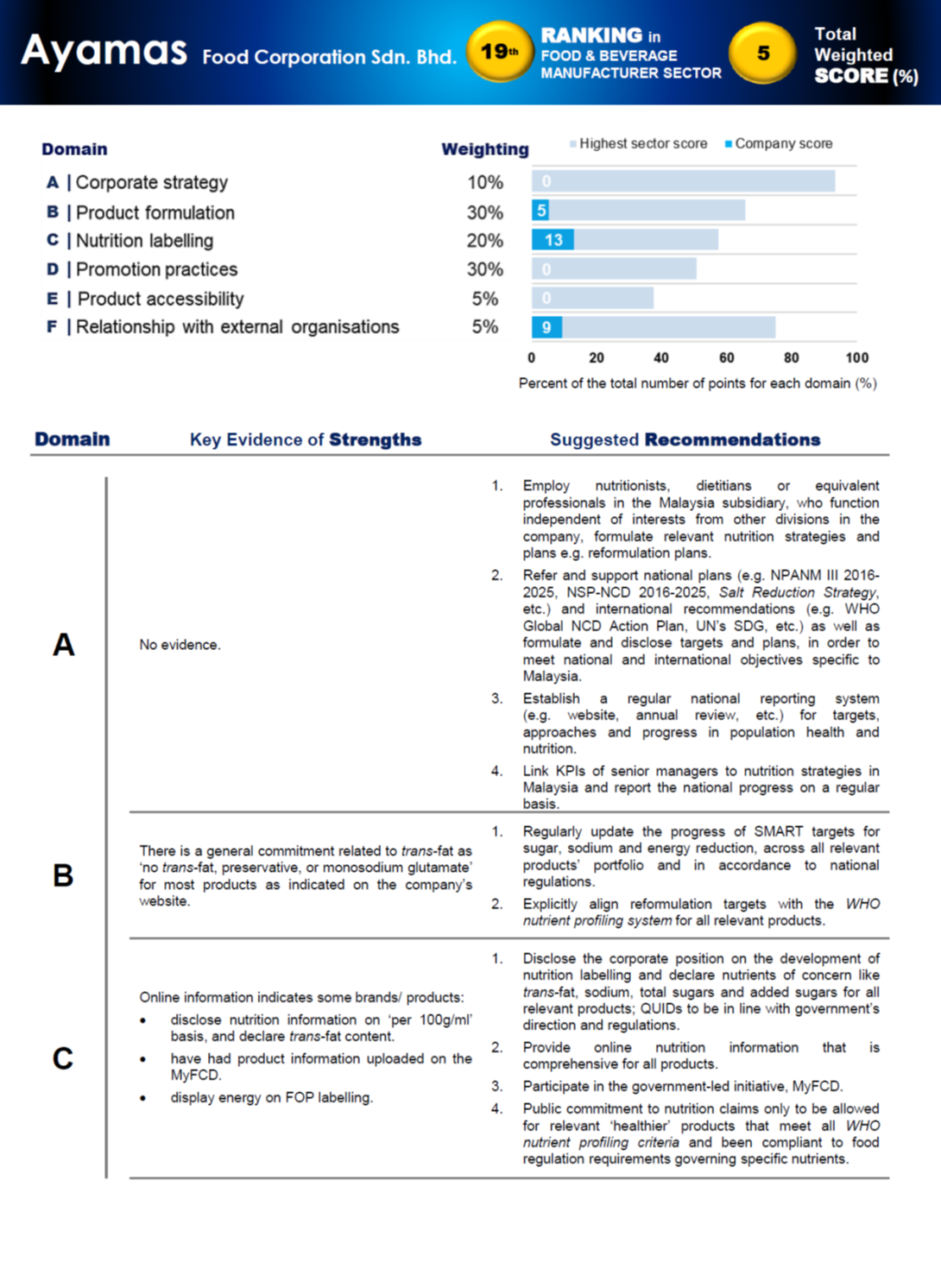
**

**
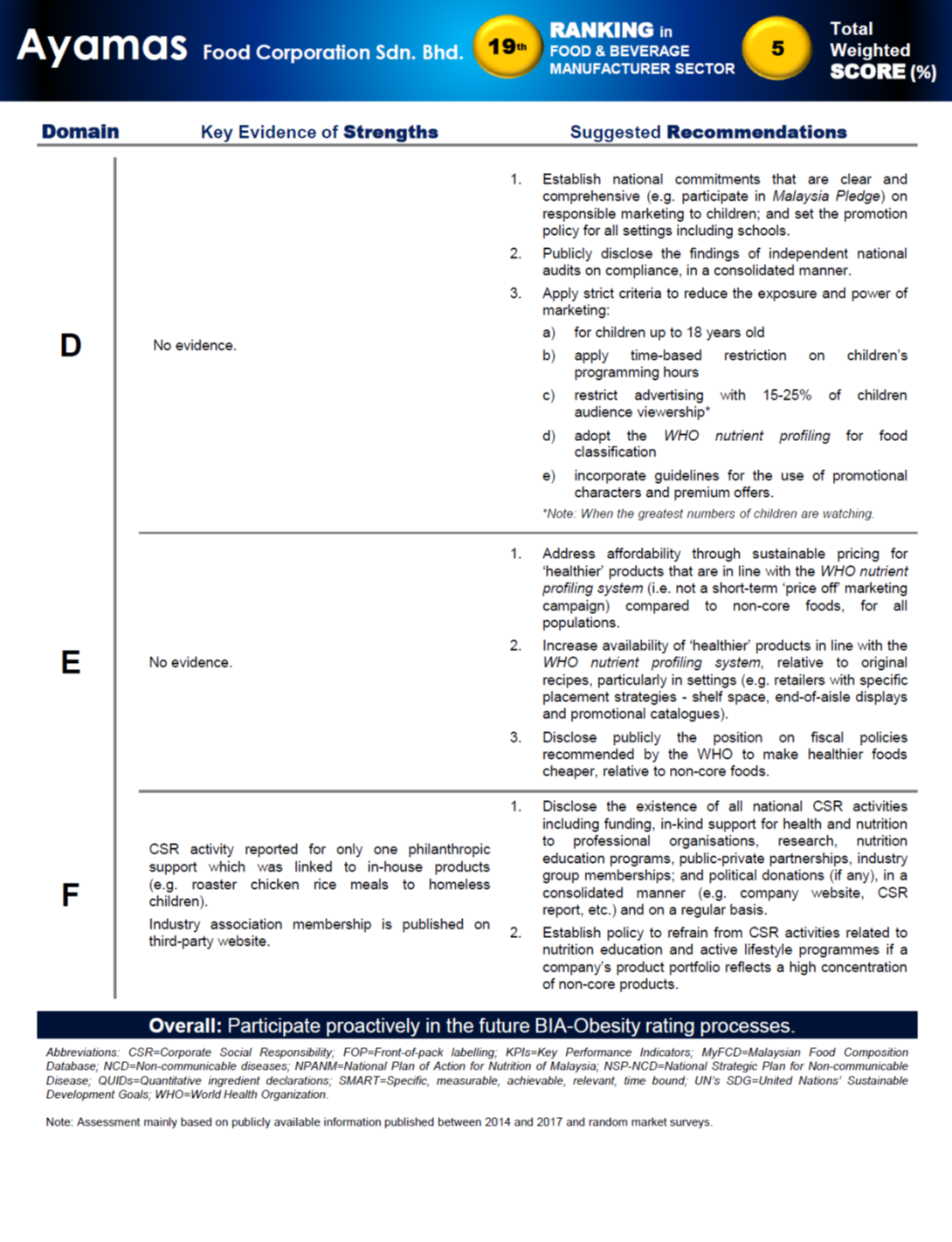
**

Supplement: Supplementary file 5 — Additional file 5 : Table S4. Example of an Individual Scorecard. A preliminary scorecard [Ayamas Food Corporation Sdn. Bhd. - a selected company] used in ‘Stage III: Findings finalisation – Feedback of preliminary findings to companies via email’.. [file 12992_2020_560_MOESM5_ESM.docx]
